# Amelioration of 5-fluorouracil-induced intestinal mucositis by *Streptococcus thermophilus* ST4 in a mouse model

**DOI:** 10.1371/journal.pone.0253540

**Published:** 2021-07-26

**Authors:** Siou-Ru Shen, Wei-Jen Chen, Hui-Fang Chu, Shiuan-Huei Wu, Yu-Ru Wang, Tang-Long Shen

**Affiliations:** 1 Center for Biotechnology, National Taiwan University, Taipei, Taiwan; 2 Syngen Biotech Co., Ltd., Tainan, Taiwan; 3 Graduate Institute of Management, Minghsin University of Science and Technology, Hsinchu, Taiwan; 4 Department of Plant Pathology and Microbiology, National Taiwan University, Taipei, Taiwan; Kyungpook National University, REPUBLIC OF KOREA

## Abstract

Intestinal mucositis is a commonly encountered toxic side effect in patients undergoing 5-fluorouracil (5-FU)-based chemotherapy. Numerous studies have shown that probiotics enable improving chemotherapy-induced intestinal mucositis, but the beneficial effects of probiotics differ depending on the strain. Therefore, in the present studies we suggest that *S*. *thermophilus* ST4 separated from raw milk may assess mucoprotective activity in 5-FU-induced intestinal mucositis. In our causal-comparative study design, fifteen mice were randomized assigned into three groups (n = 5/each group): control group, 5-FU group and 5-FU+*S*. *thermophilus* ST4 group. The control group was orally administrated saline only, and the 5-FU group was followed by intraperitoneal injection of 5-FU for 3 days after 10-day saline administration, and the 5-FU+*S*. *thermophilus* ST4 group was intragastrically subjected for *S*. *thermophilus* ST4 once per day during the whole experiment, starting from the first day of the experiment, followed by 5-FU intraperitoneal injection for 3 days after 10-day *S*. *thermophilus* ST4 pretreatment. Diarrhea score, pro-inflammatory cytokines serum levels, intestinal histopathology and short chain fatty acid were assessed. Here, we demonstrated the beneficial effects of *S*. *thermophilus* ST4 derived from raw milk against 5-FU-induced intestinal mucositis, including body weight reduction, appetite loss and diarrhea. Intrinsically, *S*. *thermophilus* ST4 effectively maintained epithelium structure in small intestines and colons as well as reduced the intestinal inflammation. Besides, *S*. *thermophilus* ST4 significantly increased the expression of acetic acid, reinforcing the muco-protective effects. In conclusion, our results demonstrate that *S*. *thermophilus* ST4 supplementation ameliorates 5-FU-induced intestinal mucositis. This suggests probiotic may serve as an alternative therapeutic strategy for the prevention or management of 5-FU-induced mucositis in the future.

## Introduction

5-FU is widely used for treatments of a range of cancers, including colorectal cancer, pancreatic cancer and breast cancers, whereas it frequently causes intestinal mucositis. Intestinal mucositis (mucosal barrier injury) characterized by a decrease in villi length and the disruption of crypt cell homeostasis is attributed to a common toxic side effect of 5-FU [1]. This side effect causes severe diarrhea, malabsorption, morphological mucosal damage and severe infection, which limits the safety and clinical applications using 5-FU as a chemotherapy. In fact, villus blunting and disrupted crypts are often seen in the small intestine upon chemotherapy due primarily to an upregulation in apoptosis and a downregulate in proliferation [2]. Similarly, in the colon, 5-FU administration significantly confers shortening the colon length, presumably the shortened colon is closely associated with severe diarrhea. [3]. Moreover, 5-FU-induced intestinal mucositis increases the production of pro-inflammatory cytokines, such as tumor necrosis factor-α (TNF-α), interleukin-1β (IL-1β), and interleukin-6 (IL-6), which are the hallmarks of mucositis inflammation [3, 4] and responsible for initiating inflammation in response to tissue injury and infection during chemotherapy. The suppression of inflammation and efficient healing abilities of the mucosa are beneficial for the maintenance of homeostasis in response to gut damage. In fact, upon an increase in mucosal repaired capability, certain cytokines involved in the repaired process had been shown to diminish the severity of intestinal mucositis both in animal models and clinical trials [5, 6]. Therefore, the strategic intervention used to block inflammatory processes or to maintain gut homeostasis are of great beneficial for 5-FU-induced intestinal mucositis.

The term probiotics is defined as “*live micro-organisms which*, *when administered in adequate amounts*, *confer a health benefit on the host*” [7]. They are commonly existed in fermented milks, yogurts and cheese or dietary supplements usually in the dehydrated form [8]. Recently, accumulative evidence supports that probiotic supplements beneficial effects for human and animal health, especially in improvements of intestinal functionalities and prevention of inflammation intestinal diseases [9]. Eventually, probiotics is known to exhibit anti-inflammatory effects through increases in the production of short-chain fatty acids (SCFAs), mainly acetate, propionate, and butyrate, stemming from carbohydrates, fibers, and polyphenols fermented by gut microbiota [10, 11]. Functionally, in the presence of SCFAs, a range of positive effects have been demonstrated, including maintenance of the normal structure, integrity and function of the intestine [12], modulation of the colonic and intracellular environment [13], and fuel for the intestinal epithelial cells, promotion of colonic epithelial cells proliferation and gene expression [14, 15].

*Streptococcus thermophilus* is a gram-positive, lactic acid production, and ovoid-shape bacterium appearing in pairs or in short chains. Taking advantage of its lactose digestion activity, *S*. *thermophilus* has been utilized to improve individuals with lactose intolerance [16] in addition to other activities such as antioxidation [17], immune modulation [18], gastrointestinal epithelium homeostasis [19], prevention of chronic gastritis [20], attenuation of diarrhea [21, 22], alleviation of ulcer and inflammation [23] and so on. Some studies have shown that the administration of *S*. *thermophilus* strain can reduced some parameters of mucositis in animal model induced by chemotherapy, such as prevent weight loss, attenuate the diarrhea and intestinal damage [24, 25]. Although *S*. *thermophilus* has been granted as a good probiotic in varied intestinal inflammatory models, for example, alleviation of colitis symptoms in a dextran sulfate sodium (DSS) model [26, 27], the beneficial effects of probiotics *S*. *thermophilus* differ in origins and/or strains. In the current study, we evaluated the mucoprotective activity of *S*. *thermophilus* ST4 in a 5-FU-induced intestinal mucositis. Our results warrant the development of probiotic supplements for chemotherapeutic side effects in related to gastrointestinal mucositis.

## Materials and methods

### Preparation of 5-FU and *S*. *thermophilus* ST4

**Fig 1:**
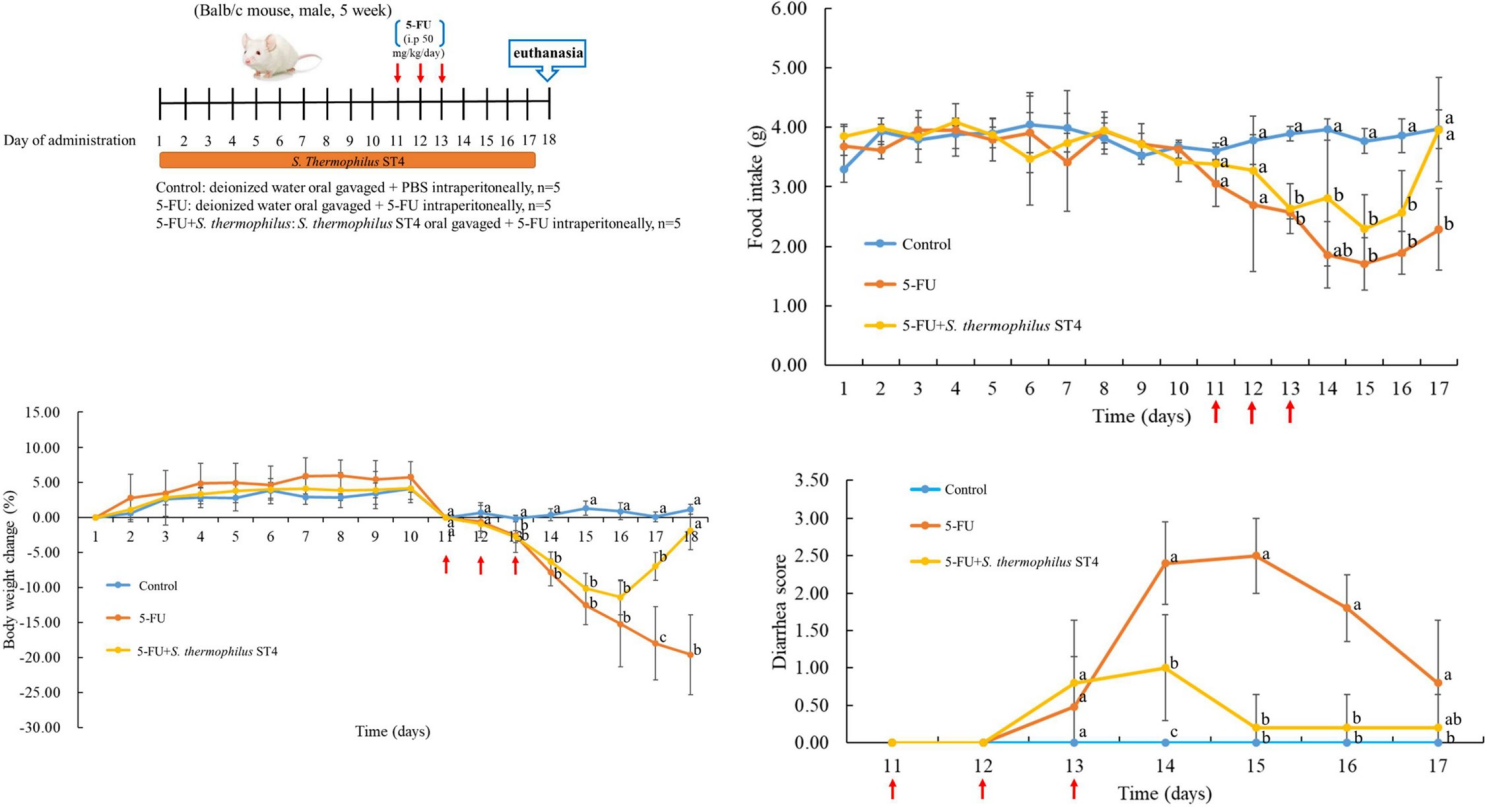
*S*. *thermophilus* ST4 attenuates 5-FU-induced intestinal mucositis. (A) Experimental design for the animal study. The group of 5-FU+*S*. *thermophilus* ST4 indicates that *S*. *thermophilus* ST4 was intragastrically subjected for pretreatment once per day for 10 days, followed by 5-FU (50 mg/kg) intraperitoneal injection once daily for 3 days, and then continuing *S*. *thermophilus* ST4 (5×10^8^ CFU/day) intragastrical administration once daily for additional 4 days. The group of 5-FU is the mice were only treated with 5-FU but no *S*. *thermophilus* ST4. Mice without any treatment of both 5-FU and *S*. *thermophilus* ST4 were as the control group. Arrows indicate the dates for the injection of 5-FU. (B) Body weight is shown as a percentage of initial body weight in a diary base. (C) Diary food intake was measured diary for each group. (D) The severity of diarrhea is scored using the four-grade scale (0–3) starting from 5-FU treatment toward sacrifice. Data are present as mean ± SD. The data with different superscripted letters are significantly different based on the one-way ANOVA (*p*<0.05).

5-FU was purchased from Sigma (St. Louis, MO, USA). The preparation of 5-FU was firstly dissolved in saline at a concentration of 5 mg/mL, and then sterile filtered through a 0.2 μm syringe filter. 5-FU was injected intraperitoneally at a single dose of 50 mg/kg/day for 3 consecutive days to cause intestine mucositis. *S*. *thermophilus* ST4 was isolated in raw milk and provided by Syngen Bio-Tech Co., Ltd. (Tainan, Taiwan). *S*. *thermophilus* ST4 was diluted in sterile water and administered by oral gavage. The mice received 100 μL of suspension containing 5×10^8^ CFU of the probiotics cocktail daily for 17 days as described in Fig 1A.

### In vivo experiments

#### Animal care

Five-week-old male BALB/cByJNarl (BALB/c) mice were purchased from the National Laboratory Animal Center (Taipei, Taiwan). The mice were housed in a climate-controlled environment (23 ± 2°C, relative humidity of 50 ± 5%) with a 12 h of light/dark cycle and allowed free access to food and water *ad libitum*. The mice adapted to the environment for 2 weeks. The animal experimental protocol used in the current study was reviewed and approved by the Institutional Animal Care and Use Committee of the National Taiwan University according to the principles of the 3Rs (Replacement, Reduction and Refinement). The experimental design tried to mimic the 5-FU-indcued mucositis by treating mice with low dosage of 5-FU along with or without *S*. *thermophilus* ST4 to evaluate the multiple effects, such as body weight change, diarrhea, inflammation, histopathology etc, of the mice on definitive mucositis in comparison with the saline control. When the loss of body weight was reached up to 20% compared to that measured at the point before the 5-FU treatment, the experimental animal was euthanized. Five mice for these three groups are enough for statistical analyses of data collected.

#### *In vivo* intestinal mucositis model

The experimental set-up is illustrated in Fig 1A and the mice were randomized to one of three groups (n = 5/group) including control group, the 5-FU-induced intestinal mucositis group, and 5-FU+*S*. *thermophilus* ST4 (5×10^8^ CFU/day) group using random number tables to achieve randomization. Intestinal mucositis was induced on the 11^th^ to 13^th^ days by intraperitoneal injection of 5-FU (50 mg/kg/day) for 5-FU group and 5-FU+*S*. *thermophilus* ST4 group. An injection of saline into mice were used as the control group. Mice were euthanized on the 18^th^ day.

Disease severity was assessed daily by measuring body weight and diarrhea status, the latter was graded based on the stool consistency: 0 (normal, normal stool or absent); 1 (slight, slightly wet and soft stool); 2 (moderate, wet and unformed stool with moderate perianal staining of the coat); and 3 (severe, watery stool with severe perianal staining of the coat) [21]. All of data were quantitative collection. For example, fecal samples from each mouse were individually, i.e. each mouse was placed a single clean cage with no bedding waiting for defacating 2–3 fecal pellets, collected, recorded and labelled in a test tube before they were stored in -80°C. There are two variables in the current study, 5-FU and *S*. *thermophilus* ST4. In fact, 5-FU treatment enables inducing the phenomenon of mucositis and *S*. *thermophilus* ST4 is the test factor in effect on ameliorating the mucositis. The amounts (concentrations) of both variables have been tested in our preliminary study (data not shown). Here, we grouped the mice into 3 groups, saline treatment, 5-FU alone, and 5-FU+*S*. *thermophilus* ST4, respectively, to evaluate the effect of *S*. *thermophilus* ST4 on 5-FU-induced mucositis. Then, the mice were sacrificed under anesthesia to collect their entire small intestines (mainly jejunum tissue) and colons (range from cecum to rectum) after removal of fact tissue and colon length (range excludes the cecum) were measured accordingly.

#### Morphology and histopathology analysis

For the assessment of pathological changes, the small intestines and colons were fixed in 10% formaldehyde solution and embedded in paraffin. Sections with 3–5 μm in thickness were cut, deparaffinized, rehydrated, stained with hematoxylin and eosin (H&E). The morphological alteration and inflammatory cell infiltration were examined under a light microscope (DL 882096, LWScientific, Inc., Georgia, USA). The photos were taken at a final magnification of 100× and 400×.

#### Immunohistochemical staining of the small intestine

Sections (3 μm thick) were cut and mounted on a glass slide and followed by deparaffinization in xylene, rehydration in a graded ethanol series, antigen retrieval using microwave and then endogenous peroxidase inactivation in immersing specimens in 3% H_2_O_2_. To perform immunostaining, slides were incubated at 4°C for overnight with rabbit polyclonal anti-F4/80 antibody (Abcam, cat.# ab6640) at 1: 1000 dilution and followed by another incubation with HPR-conjugated anti-rabbit secondary antibody (Jackson lab) at 1:2000 dilution at room temperature for 1 hour. After washes, sections were developed with diaminobenzidine (DAB) and counterstained with hematoxylin, and then dehydrated in a graded alcohol series, cleared in xylene, and placed with coverslips. Positive F4/80 cells were counted using Image J^®^ software (Rasband, W.S., ImageJ, U. S. National Institutes of Health, Bethesda, MD, USA).

#### Pro-inflammatory cytokines analysis

To quantify cytokines, blood was first collected from the heart immediately after mice were sacrificed. Blood samples were then centrifuged to obtain serum. Concentrations of pro-inflammatory cytokines (TNF-α, IL-1, and IL-6) in serum were measured using corresponding enzyme-linked immunosorbent assay kits (ELISA; Thermo Fisher Scientific, Inc.) according to the manufacturer’s instruction. Results are expressed as pg/mL.

#### Short-chain fatty acids (SCFAs) analysis of feces

SCFAs were quantified according to the previously published description [28]. Briefly, fresh fecal samples were collected and homogenized with cold saline (NaCl 0.9%, w/v) at a ratio of 1:10 (w/v) prior to centrifugation for 10 min at 1,000 *g*. Collected supernatants were acidified with 20 μL of 50% (w/v) sulfuric acid containing isocaporic acid as an internal standard. Then, SCFAs were mixed and extracted by diethyl ether. The extracted samples were directly injected into the GC column (Agilent J and W HP-INNO Wax GC Column, 30 m, 0.25 mm id, 0.25 μm film thickness). The GC system consisted of an Agilent 7890A (Agilent Technologies, Palo Alto, CA, USA), equipped with a flame ionization detector. Helium as carried gas was used for the separation at a flow rate of 7 mL/min. One μL of each sample was injected with an injector temperature of 140°C and the detector temperature 250°C. The conditions were as follows: oven temperature, initially held at 80°C for 1 min and then raised to 140°C at a rate of 20°C/min, then held at 140°C for another 1 min, and raised again to 220°C at a rate of 20°C/min, and lastly held at 220°C for 2 more mins.

### Statistical analysis

All data were analyzed by GraphPad Prism 6.0 software and presented as means ± SD from the indicated number of independent experiments. Comparisons of statistical significance between experimental groups were determined by one-way analysis of variance (ANOVA) using SPSS for Windows (version 12.0) of variance followed by Duncan’s multiple range method.

## Results

### Effect of *S*. *thermophilus* ST4 treatment on 5-FU-induced body weight loss, food intake and diarrhea

To evaluate the protective effect of *S*. *thermophilus* ST4 on 5-fluorouracil-induced intestinal mucositis, mice were oral gavage with or without *S*. *thermophilus* ST4 prior to 5-FU-induced intestinal mucositis and monitored their body weight loss, food intake and diarrhea dairy (Fig 1A). On contrary to the control group, the 5-FU group mice resulted in significantly decreased body weight (about 20%), food intake (about 50%), diarrhea (score from 0 up to 2.5), reluctance to move and hair loose (data not shown). Nevertheless, we found that the administration of *S*. *thermophilus* ST4 apparently ameliorated the aforementioned clinical symptoms of 5-FU-induced intestinal mucositis toward no significant difference compared to the control group as shown in Fig 1B–1D. Here, the daily evaluation of intestinal mucositis induced by 5-FU was followed by weight reduction throughout the experimental period. Compared to the control group, gradual body weight loss was observed in mice treated with 5-FU, and the mean body weight (relative mean body weight change (%) was recorded daily and expressed as mean from baseline at day 11^th^ = 0%) was reduced 19.59% of initial body weight on the 18^th^ day. Intricately, our data showed that the *S*. *thermophilus* ST4 obviously rescued 5-FU-induced impairment in the severity of 5-FU-induced intestinal mucositis resulting from the reductions in body weight loss only 1.85% as shown in Fig 1B. In addition, the food intake was irreversibly by 5-FU+*S*. *thermophilus* ST4 compared to the 5-FU treated group reduced by 50% (Fig 1C), reaching to the same as the control group. Diarrhea was assessed by using the Bowen’s score system. We classified diarrhea into four grades according to the stool consistency: 0, normal stool; 1, slightly wet and soft stool indicating mild diarrhea; 2, wet and unformed stool indicating moderate diarrhea; and 3, watery stool indicating severe diarrhea. accordingly, we observed and recorded the diarrhea score of each mouse starting on the day of 5-FU treatment. As shown in Fig 1D, diarrhea scores exhibited a significant reduction and recovery in the 5-FU+*S*. *thermophilus* ST4 treatment compared to the 5-FU-induced group from the most severe day 14^th^ to day 15^th^, and the mean diarrhea score changed from 1.0 to 0.1 and from 2.5 to 2.50, respectively (Fig 1D). Taken together, these data clearly present the potential effect of *S*. *thermophilus* ST4 on protecting the 5-FU-induced intestinal mucositis in a mouse model.

### Effects of *S*. *thermophilus* ST4 on 5-FU-induced intestinal mucositis

**Fig 2:**
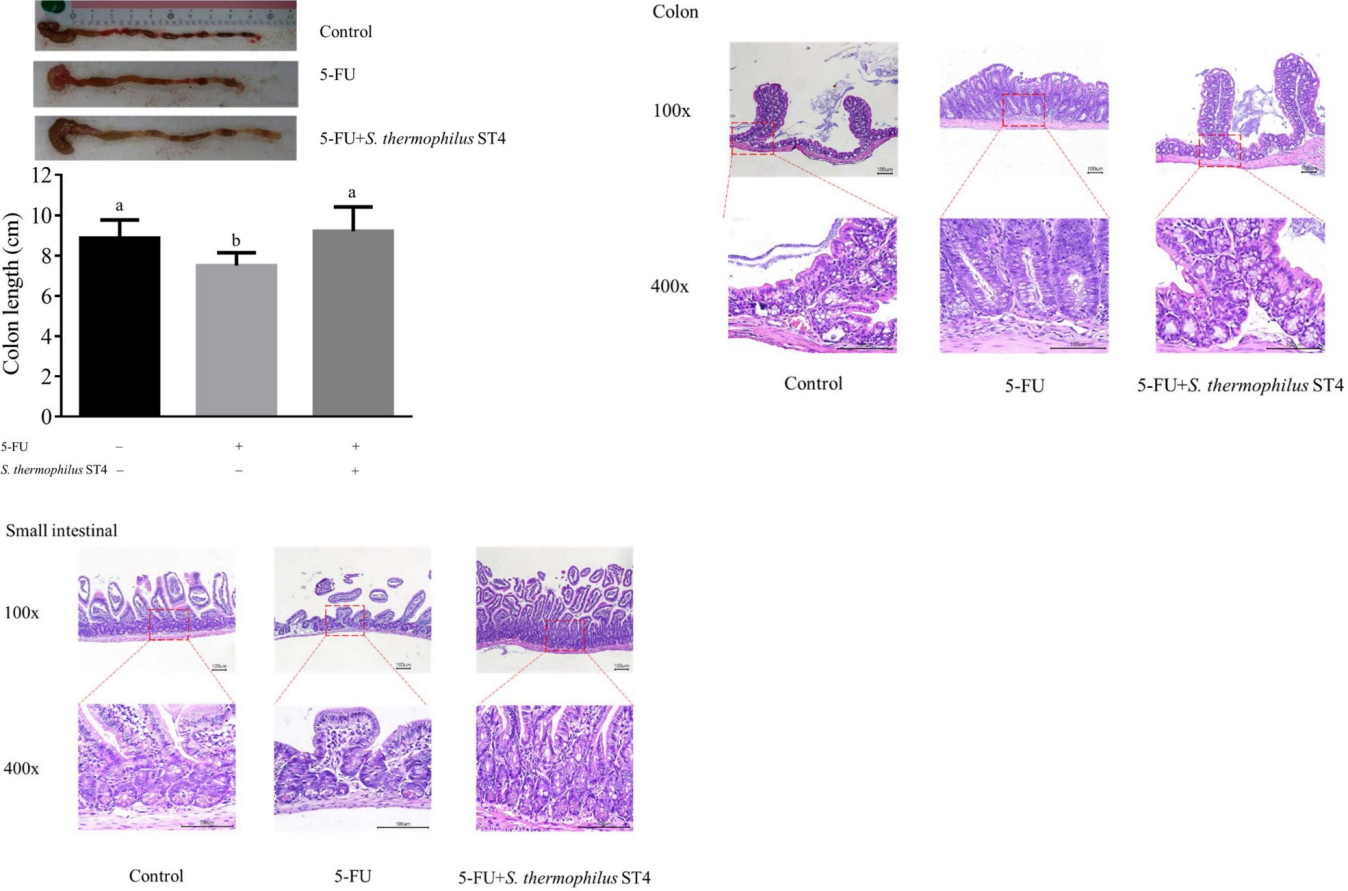
Histopathological examination of 5-FU-induced intestinal mucositis with or without *S*. *thermophilus* ST4 treatment. Three groups of mice as indicated were sacrificed according to the experiment design subjected for measuring (A) colon length and (B and C) for H&E staining of the small intestine and colon after excised, sectioned, and then stained, respectively. The representative images (100X and 400X magnifications) of each group are presented. Data are present as mean ± SD. The data with different superscripted letters are significantly different based on the one-way ANOVA (*p*<0.05).

To further evaluate histopathological characteristics, we firstly examined the length of the colon among the different treated groups. In our experiments, the average colon length (7.5 cm) of 5-FU treated mice was significantly shortened compared to that (8.9 cm) of the control group (Fig 2A), whereas the colon length shorten was markedly alleviated and remained at the average of 9.2 cm by treatment with *S*. *thermophilus* ST4 even under 5-FU treated condition. Furthermore, we examined the change of small intestines treated with 5-FU followed with or without *S*. *thermophilus* ST4 at the microscopic level (Fig 2B and 2C). Morphological analyses indicated that the villus heights to crypts depths of 5-FU treated mice were dramatically shortened in comparison with the control mice, while the reduction of small intestine villus heights and crypts depths was markedly alleviated by treatment with *S*. *thermophilus* ST4 as shown in Fig 2B. Our results suggested that the 5-FU-induced mucositis in small intestines could be prevented by 5-FU+*S*. *thermophilus* ST4. Likewise, the obvious differences were observed in colon morphology after the induction of mucositis compared to the control group and 5-FU group. 5-FU resulted in the epithelial and crypt damage in colon tissues, and the crypt depth was dramatically decreased by the mucositis as shown in Fig 2C. As expected, 5-FU+*S*. *thermophilus* ST4 administration exhibited protective effects on colon damage.

### *S*. *thermophilus* ST4 suppressed 5-FU-induced pro-inflammatory cytokines

**Fig 3:**
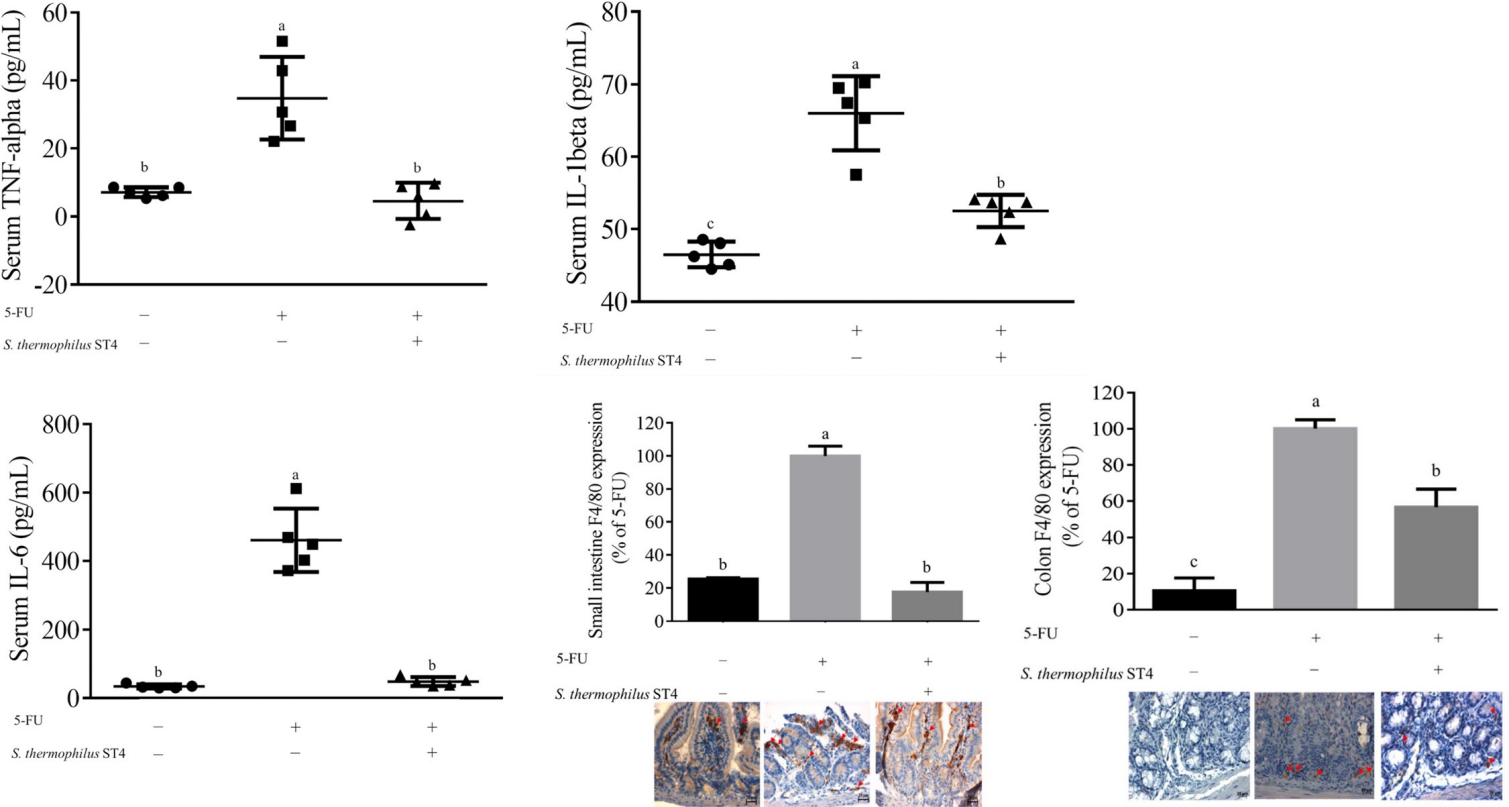
Reduction of the inflammatory effect by *S*. *thermophilus* ST4 in the 5-FU-induced intestinal mucositis mouse model. Despite of elevated expressions of TNF-α (A), IL-1β (B) IL-6 (C) and F4/80 (a marker for macrophage, D and E) induced by 5-FU, *S*. *thermophilus* ST4 enabled significantly diminishing the increase of these inflammatory factors in serum or in the intestine (D) and the colon (E) of the 5-FU-induced mucositis mice. Data are present as mean ± SD. The data with different superscripted letters are significantly different based on the one-way ANOVA (*p*<0.05).

To understand the mechanistic nature of *S*. *thermophilus* ST4 suppressed 5-FU-induced mucositis, we examined expression of inflammatory factors in the presence or absence of *S*. *thermophilus* ST4 in a 5-FU-induced mouse model. Indeed, inflammation is known to be involved in the pathogenesis of mucositis, and the pro-inflammatory cytokines are considered as important factors and potential targets for treatment of mucositis. As shown in Fig 3A–3C, in 5-FU-induced mice, the pro-inflammatory cytokines concentrations of TNF-α (34.78 pg/mL), IL-1β (66.02 pg/mL) and IL-6 (460.68 pg/mL) were significantly increased in comparison with the control group (TNF-α at 7.10 pg/mL, IL-1β at 46.52 pg/mL and IL-6 at 34.17 pg/mL), respectively. Similar to the control group, the 5-FU+*S*. *thermophilus* ST4 group significantly reduced the concentrations of TNF-α, IL-1β and IL-6 at 4.52, 52.51, and 48.53 pg/mL, respectively, compared to the 5-FU group. In agreement with the above result, we also found that the *S*. *thermophilus* ST4 group gave rise to less macrophage infiltrations in the small intestine and the colon as shown in Fig 3D and 3E, in compared to those in 5-FU- induced mice, from 56.52% to 17.58%. To be noted, 5-FU treatment impaired mucosal epithelium and disrupted crypt-villus structures, which was accompanied with increase cellular infiltration and macrophage (F4/80 stain) aggregation. in contrast, 5-FU+*S*. *thermophilus* ST4 administration exhibited protective effects on intestinal mucositis.

### Effects of SCFAs production by the *S*. *thermophilus* ST4 on 5-FU-induced intestinal mucositis

**Fig 4:**
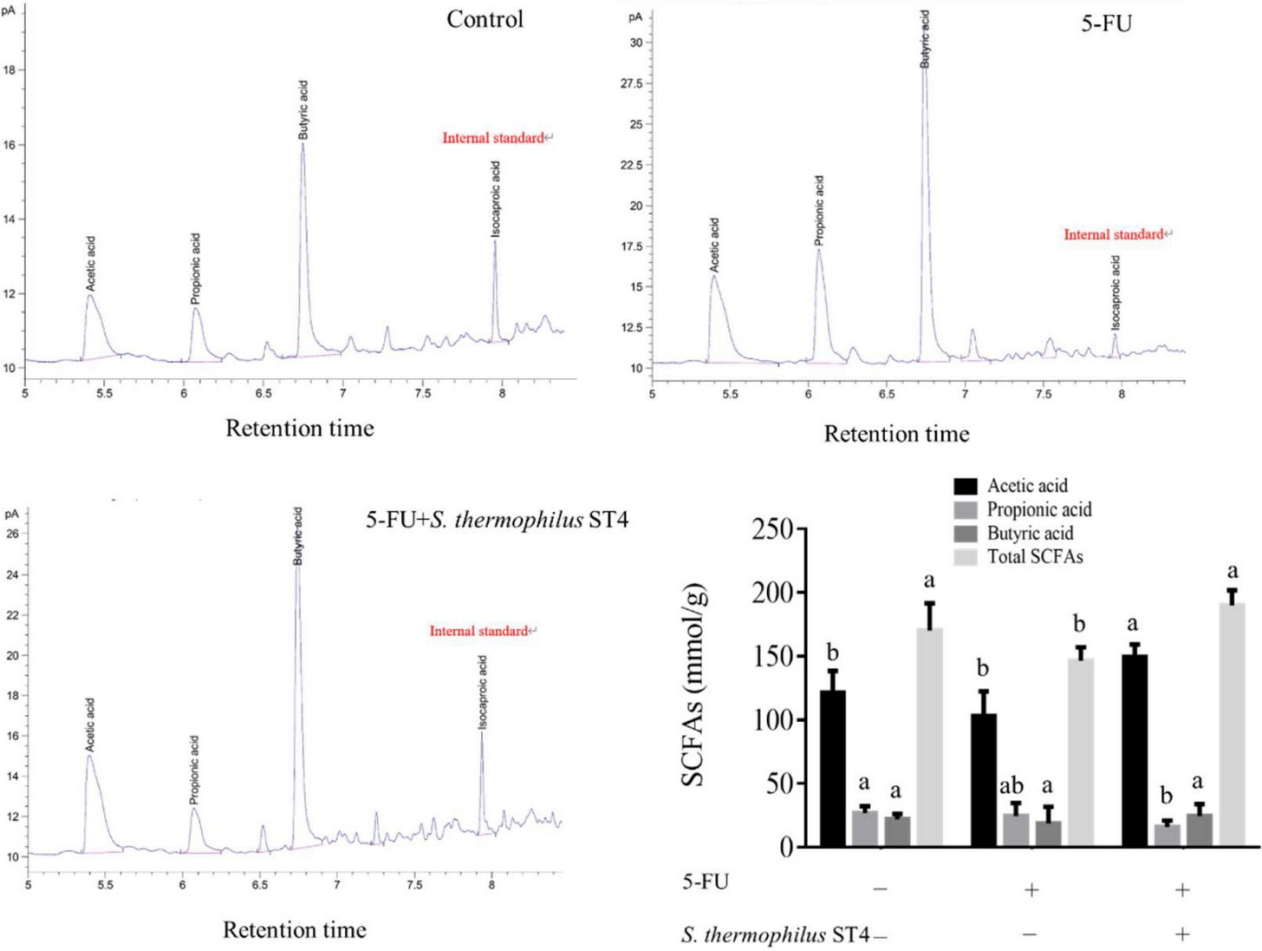
*S*. *thermophilus* ST4 promotes SCFAs production of the 5-FU-induced mouse. (A-C) HPLC profiles and (D) concentration (μmol/g) of SCFAs in feces collected from three groups. Isocaproic acid as an internal standard in HPLC. Data are present as mean ± SD of 5 mice. The data with different superscripted letters are significantly different based on the one-way ANOVA (*p*<0.05).

To elucidate the essential metabolite in correlation with the anti-inflammatory effect of the *S*. *thermophilus* ST4 on 5-FU-induced intestinal mucositis in the mouse model, we investigated the fecal concentrations of various short-chain fatty acids among the different groups. Here, we further revealed a higher concentration of acetic acid in the stool of the 5-FU+*S*. *thermophilus* ST4 group (78.86 mmol/g) than that in the control group and 5-FU group (71.28 mmol/g and 70.69 mmol/g, respectively) as shown in Fig 4, implicating this short-chain fatty acid rather than other SCFAs, including propionic acid and butyric acid, critical for the protective effect of *S*. *thermophilus* ST4 on intestinal mucositis.

## Discussion

In this present study, we recap 5-FU injected mice resulting in body weight loss, reduced food intake, and diarrhea in our experimental model, which is related to the negative effects of intestinal mucositis as previous reports [3, 29, 30]. Apparently, we showed that *S*. *thermophilus* ST4 enabled attenuating the 5-FU-induced body weight loss indicated by the improvement in food intake. Additionally, *S*. *thermophilus* ST4 also alleviated the diarrhea of 5-FU-indcued mucositis mice. A previous study evaluated *S*. *thermophilus* strain (NCIMB 41856) is concluded to promote maintenance of mucosal barrier function and, therefore, indirectly reduce immune stimulation and inflammation in a DSS mouse model to ameliorate signs of colitis in an iron-rich condition [27]. However, our current study does obviously see the additional differences in histopathology and cytokine production between probiotics treatment group or non-treatment group in a 5-FU-induced mucositis mouse model. We performed the effect of 5-FU on *S*. *thermophilus* ST4 cytotoxicity in vitro, none of which induced cytotoxicity in the *S*. *thermophilus* ST4 that we tested [S1 Appendix]. In addition, we tried to allow the *S*. *thermophilus* ST4 colonized on the intestine before 5-FU-induced mucositis to provide a protective effect. In contrast, the Bailey et al administrated DSS to induce colitis at the beginning in combination with treatment of *S*. *thermophilus* strain (NCIMB 41856). Thus, these studies provide more beneficial effects of *S*. *thermophilus* strains on mucositis while the probiotics are applied to the animal as earlier.

The intestinal epithelium is a single-cell layer composing the largest and most important barrier whose primary function is to assist in absorption of nutrients across the epithelial lining in addition to maintaining a physical barrier against the external environment [31]. Efficient absorption is enhanced within the small intestine via finger-like projections called villi, which greatly augment the surface area in contact with luminal contents [32]. The intestinal barrier is maintained by epithelial cells joined by tight junctions on their lateral boarders, thus the selective passage of luminal contents across the cell being formed [32]. To be noted, crypts are flask-like structures around the base of villi containing proliferative units necessary for maintaining epithelial integrity of throughout the intestine [32]. In contrast, intestinal shortening is a phenomenon which is depicted both in experimental colitis model [33] and chemotherapy induced mucositis [34], suggesting destruction or inflammation in intestine. For example, 5-FU-induced mucositis effect on intestinal morphology is often characterized by decreased crypt length, blunting and fusion of villi, enterocytes hyperplasia and increased apoptosis [35], thereby resulting in the disruption of mucosal integrity as well as the shape changes of villus and crypt parameters. The gastrointestinal crypt epithelium is particularly vulnerable to chemotherapy drug toxicity with symptoms including nausea and vomiting, abdominal pain, distension, and diarrhea [36]. Nevertheless, studies have reported that the oral administration of *S*. *thermophilus* TH-4 at a dose of 10^9^ CFU/mL, partially attenuated methotrexate-induced small intestinal damage [24]. Moreover, [25] showed that live TH-4 and supernatant partially normalized mitotic count and histological severity score while 5-FU-induced intestinal mucositis.

Morphologically, the broken villi in the small intestine and colon were dramatically increased upon the induction of intestinal mucositis in the current study, while the loss of crypt structure was occurred in colon segments. We demonstrated that the structures of villus and crypt in small intestine and colon were significantly protected by *S*. *thermophilus* ST4 treatment at 5×10^8^ CFU/day for 17 days, in which *S*. *thermophilus* ST4 might remain mucosal growth during chemotherapy.

Rather than the direct injury to intestinal basal stem cells, 5-FU-induced intestinal mucositis also leads to a consequence of complex biological events, including reactive oxygen species (ROS) generation, immune cells infiltration, and pro-inflammatory cytokines over-production [37, 38]. Pro-inflammatory cytokines, such as TNF-α, IL-1β, and IL-6, play a role in amplifying the severity of chemotherapy-induced intestinal mucositis [39]. In fact, TNF-α is a key factor in the caveolin-1-mediated internalization of occluding, which elevates gut permeability [40]. Besides, TNF-α is a very potent matrix metalloproteinases produced by neutrophils and activated macrophages in contribution to the epithelial ulceration and sub-mucosal destruction [41]. Numerous studies have revealed elevated productions of the inflammasome-dependent cytokines IL-1β and IL-18 during clinical and experimental chemotherapy-induced mucositis [42]. Consistently, IL-1β enhances intestinal mucositis pathogenesis by triggering apoptosis of intestinal crypt epithelial cells via p53-mediated upregulation of p21 and p27 [43]. Moreover, [44] substantiated the importance of IL-6 in 5-FU-induced small intestine injury using an IL-6^-/-^ mouse model, also indicating the caspase-3-dependent pathway is involved in IL6-mediated apoptosis in the ileum and colon in response to 5-FU treatment. On the other hand, IL-6 is recognized as an important mediator of gut dysfunction in IBD [45] in concordance with an increase in IL-6 signaling often observed in the inflamed mucosa of IBD [46]. In fact, expressions of TNF-α, IL-1β and IL-6 were also found to be increased in response to the 5-FU treatment, indicating that inflammatory cytokines play a key role in the pathogenesis of mucositis induced by chemotherapy and radiotherapy. Therefore, it is believed that TNF-α, IL-1β and IL-6 are involved in mucositis and have been the targets of inhibition [4]. Herein, we showed that TNF-α, IL-1β and IL-6 were significantly increased following 5-FU treatment, while these increases were attenuated by *S*. *thermophilus* ST4 treatment. In combination of mucositis phenotypes, *S*. *thermophilus* ST4 serving as a regimen enables attenuating the severity of intestinal mucositis induced by 5-FU through the inhibition of pro-inflammatory cytokines expression.

Numerous intestinal diseases are characterized by immune cell activation in association with the detriment of epithelial barrier function. Based on the model of gastrointestinal mucositis reported by [47], infiltrated phagocytes, such as macrophages and neutrophils, at inflamed sites, are thought to be responsible for the formation of ROS, which can subsequently alter the localization of tight junction components such as ZO-1 and occluding [48]. In fact, macrophage infiltration is a common feature of inflammation in the chemotherapy-induced intestine [4]. In consistence with the above, severe damages in small intestine and colon manifested by villus deformation, loss, and atrophy were accompanied with enhanced macrophage infiltration in mice by 5-FU treatment. In contrast, *S*. *thermophilus* ST4 enables decreasing the infiltration of macrophage into distal mucosa and protecting the structural integrity of small intestine and colon tissue.

SCFAs (short chain fatty acids) can be resulted from bacterial fermentation in the intestine to provide energy for colonic mucosal epithelial cells and essential for the development and mediation of the intestinal barrier function [49, 50]. We showed that *S*. *thermophilus* ST4 significantly increased fecal acetic acid concentration, one of common and functional SCFAs. Studies have revealed that acetate enables inhibiting fat accumulation in adipose tissue and then results in suppression of metabolic inflammation via insulin signaling in adipocytes in rodents [51]. In addition, activation of G-protein-coupled receptor 43 (GPR43) by acetate markedly protects against gut inflammation in the model of colitis [52]. Reportedly, studies have observed that *Bifidobacteria* can protect the host against lethal infection via the production of acetate [53]. [54] revealed that acetic acid increased DNA synthesis in a human colonic epithelial cell line from adenocarcinoma (LS-123 cells) and a non-transformed small intestinal cell line from germ-free rats (IEC-6 cells).

There are several limitations in this study. One limitation is that we focused on the histological effects of small intestines and colon, other parts of the gastrointestinal tract such as stomach specimens were not examined. Also this study did not address the possible mechanisms by which *S*. *thermophilus* ST4 might exert their beneficial outcomes to sustain tight junction proteins and transepithelial electrical resistance. These areas should be investigated in the future.

On the other hand, further studies should also focus on the exploration of *S*. *thermophilus* ST4-derived compounds in effect on tight junction expression and intestinal permeability should be conducted to better elucidate the mechanisms underlying maintenance of intestinal barrier functions since that we observed a major effect on ameliorating diarrhea in response to the 5-FU treatment. More clinical works are needed to demonstrate the beneficial effects of *S*. *thermophilus* ST4 in the management of 5-FU-induced intestinal mucositis.

## Conclusion

**Fig 5:**
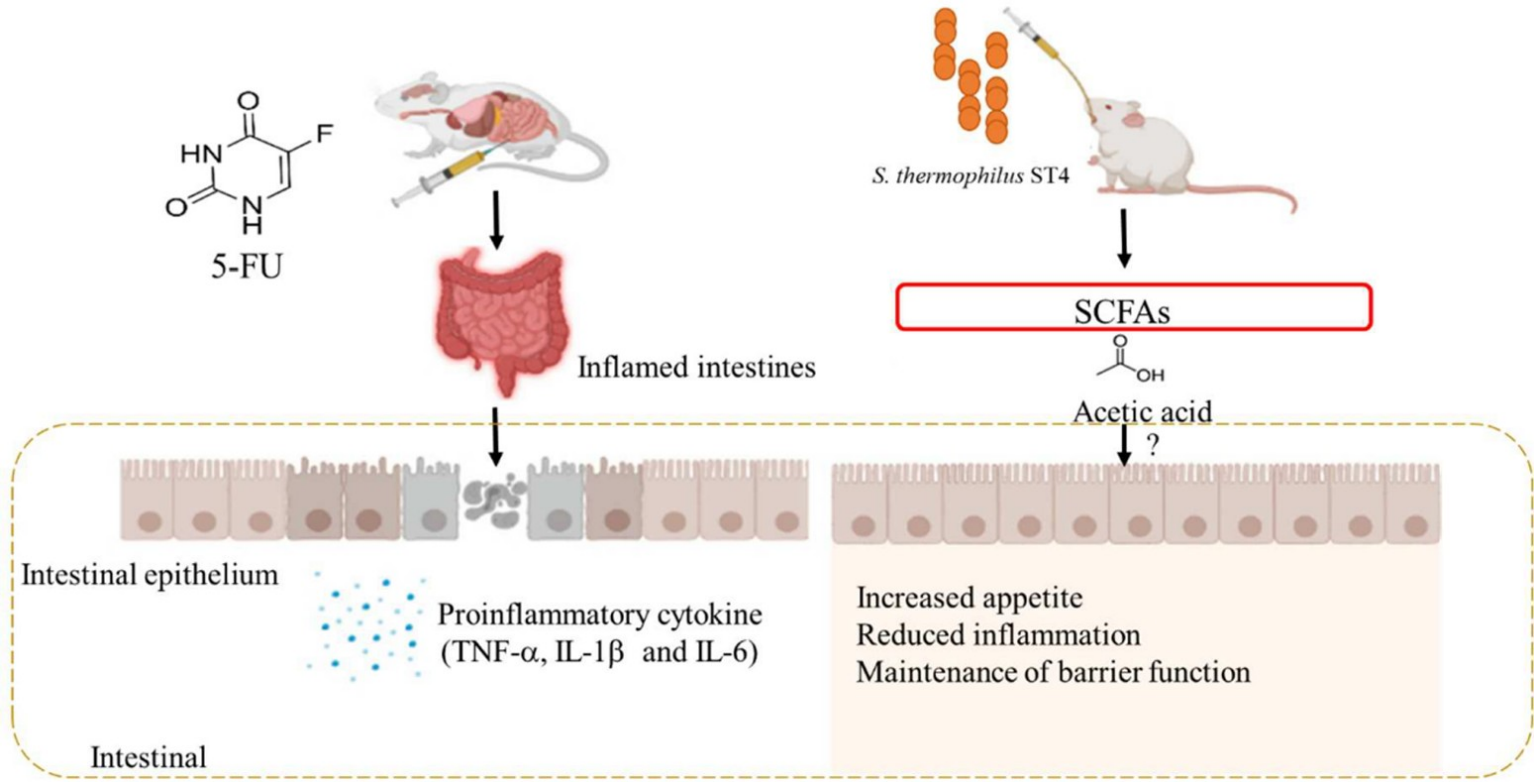
A schematic model for *S*. *thermophilus* ST4 attenuating 5-FU-induced intestinal mucositis. Elevation of SCFAs, for example acetic acid, in intestine enables diminishment of inflammation as well as maintenance of barrier function in intestine and colon, therefore resulting in increased appetite and in reducing diarrhea in the 5-FU-induced intestinal mucositis.

The present work showed that oral administration of probiotic *S*. *thermophilus* ST4 enables attenuating 5-FU-induced intestinal mucositis. The *S*. *thermophilus* ST4 markedly improved the clinical complications such as weight loss, food intake and diarrhea score. Furthermore, the *S*. *thermophilus* ST4 treatment significantly mitigated the histological damage compared to the 5-FU-induced intestinal mucositis. Similarly, the biochemical changes such as inflammatory cytokines such as TNF-α, IL-1β and IL-6 were markedly alleviated attenuated by the *S*. *thermophilus* ST4 treatment. Furthermore, acetic acid in SCFAs were significantly enhanced by *S*. *thermophilus* ST4. In summary, the present results speculated that the positive effect of *S*. *thermophilus* ST4 is partially if not all attributed by an increase in the content of acetic acid in correlation with maintenance of inflammatory homeostasis as well as preservation of intestinal permeability (Fig 5). Based on these findings, our results indicated that oral administration of probiotic *S*. *thermophilus* ST4 can ameliorate 5-FU-induced intestinal mucositis in a mouse mucositis model. Accordingly, it is of indicative that probiotics may be a potential alternative therapeutic strategy for the prevention or management of 5-FU-induced intestinal mucositis in the future. More clinical works are required to demonstrate the beneficial effects of *S*. *thermophilus* ST4 and elucidate the correct dosing regimens in the management of 5-FU-induced intestinal mucositis.

## Supporting information

S1 Appendix The effect of 5-FU on *S*. *thermophilus* ST4 cytotoxicity. The 5-FU did not apparently cause cytotoxicity on *S*. *thermophilus* ST4 at the test concentration of 0.5~10 μM.
(DOCX)

S1 Table The effect of 5-FU on *S*. *thermophilus* ST4 cytotoxicity. (TIF)

## Abbreviations

5-FU: 5-fluorouracil
TNF-α: tumor necrosis factor-α
IL-1β: interleukin-1β
IL-6: interleukin-6
SCFAs: short-chain fatty acids
DSS: dextran sulfate sodium
IBD: inflammatory bowel disease
ROS: reactive oxygen species
GPR43: G-protein-coupled receptor 43
