# Supplementary material for: Amelioration of 5-fluorouracil-induced intestinal mucositis by Streptococcus thermophilus ST4 in a mouse model
Source: PLoS One. 2021 Jul 26;16(7):e0253540. doi: 10.1371/journal.pone.0253540 (PMC8312939; doi:10.1371/journal.pone.0253540)
Supplement: S1 Appendix — The 5-FU did not apparently cause cytotoxicity on S. thermophilus ST4 at the test concentration of 0.5~10 μM. (DOCX) [file pone.0253540.s001.docx]

**Effect of 5-FU on *Streptococcus* *thermophilus* ST4 cytotoxicity**

**Material and method**

5-FU was purchased from sigma (St. Louis, MO, USA). The preparation of 5-FU was dissolved in phosphate buffer (0.2 M, pH 7.4) and then sterile filtered through a 0.2 μm syringe filter. The *S. thermophilus* ST4 were provided by Syngen Bio-Tech Co., Ltd. (Tainan, Taiwan). The stock cultures of *S. thermophilus* ST4 to the stationary phase in De Man, Rogosa, Sharpe (MRS) agar.

**Cytotoxicity test**

0.1 mL of the 5-FU (0.5-10 μM/plate), 0.6 mL phosphate buffer (0.2 M, pH 7.4) and 0.1 mL of *S. thermophilus* ST4. The serial dilutions were immediately made with phosphate buffer, and then 1 mL of the aliquot was mixed with nutrient agar. After incubation at 37℃ for 48 hrs, the number of colonies was counted. A toxicity effect was confirmed if the standard plate count of the tested compound was lower than that of control (without adding tested extract).

**Result**

The experimental results showed that 5-FU did not apparently cause cytotoxicity on *S. thermophilus* ST4 at the test concentration of 0.5~10 μM. According to the results of the cell model, a cytotoxic effect was occurred toward intestinal cells IEC-6 cells resulting in reduction of the cell survival rate by 50% while 5-FU being at a concentration as low as 3 μM. Therefore, we speculated that 5-FU obviously damages intestinal epithelial cells but not *S. thermophilus* ST4 in our experimental animal setting. The less impairment of 5-FU on S. *thermophilus* ST4, allowing ST4 remaining adherent onto the intestinal mucosa to present its protective functions leading to attenuate 5-FU-induced intestinal mucosa inflammation-related pathological symptoms.
