# Supplementary figures and images for: Amelioration of 5-fluorouracil-induced intestinal mucositis by Streptococcus thermophilus ST4 in a mouse model
Source: PLoS One. 2021 Jul 26;16(7):e0253540. doi: 10.1371/journal.pone.0253540 (PMC8312939; doi:10.1371/journal.pone.0253540)

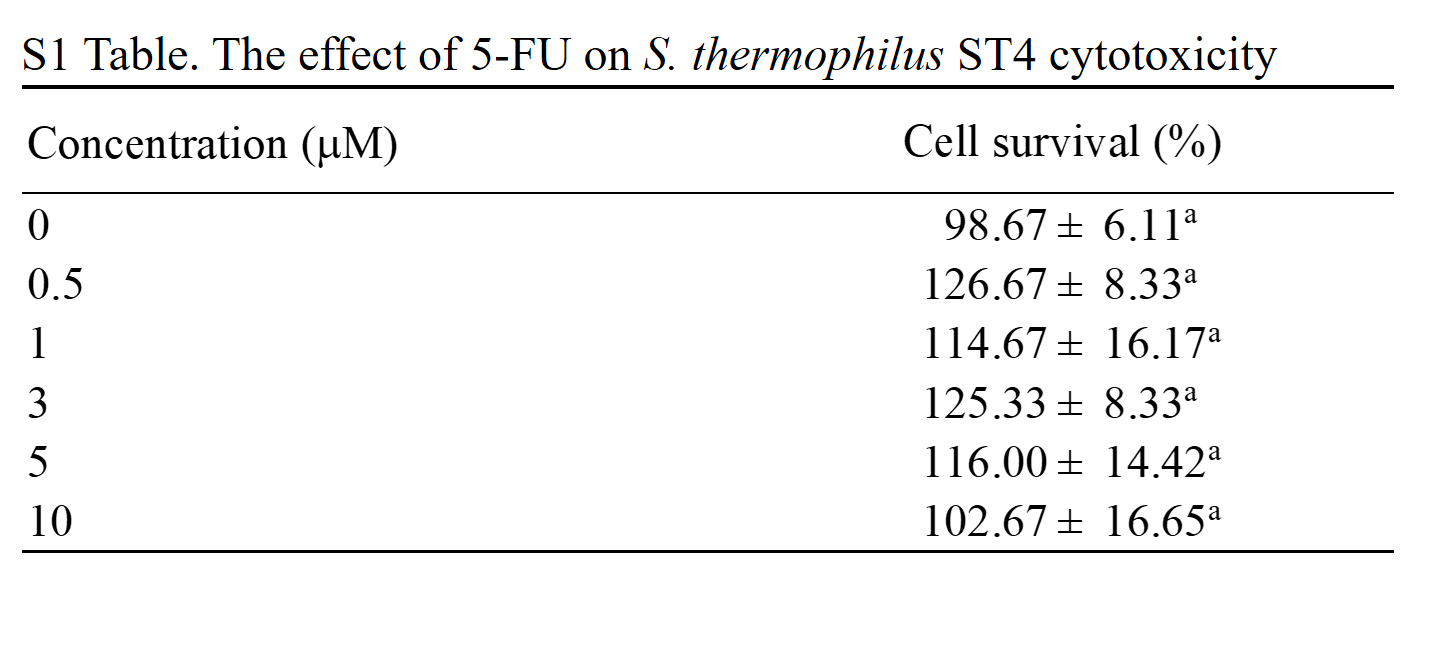

Supplement: S1 Table — (TIF) [file pone.0253540.s002.tif]
